# Hepatitis C prevalence and quality of health services among HIV-positive mothers in the Democratic Republic of the Congo

**DOI:** 10.1038/s41598-022-05014-3

**Published:** 2022-01-26

**Authors:** Peyton Thompson, Christian Mpody, Wesley Sayre, Clare Rigney, Martine Tabala, Noro Lantoniaina Rosa Ravelomanana, Fathy Malongo, Bienvenu Kawende, Frieda Behets, Emile Okitolonda, Marcel Yotebieng, CQI-PMTCT study team, Godelive Aitikalema, Ali Alisho, Elysée Bayayana, Fabrice Bumwana, Pierre Dianzenza, Jean Claude Dinanga, Georges Kihuma, Willy Lukumu, Fidèle Lumande, Zouzou Masevo, Fanny Matadi, Rachel Mushiya, Marie Therèse Mwela, José Nlandu, Pearl Tenatena, Marie Tshibuabua

**Affiliations:** 1 Division of Infectious Diseases, Department of Pediatrics, University of North Carolina, Chapel Hill, NC USA; 2 Division of Epidemiology, College of Public Health, The Ohio State University, Columbus, OH USA; 3 School of Medicine, University of North Carolina, Chapel Hill, NC USA; 4 College of Health Sciences, Cleveland State University, Cleveland, OH USA; 5 School of Public Health, University of Kinshasa, Kinshasa, Democratic Republic of Congo; 6 Department of Epidemiology, Gillings School of Global Public Health, University of North Carolina at Chapel Hill, Chapel Hill, NC USA; 7 Division of General Internal Medicine, Department of Medicine, Albert Einstein College of Medicine, Bronx, NY USA

**Keywords:** Hepatitis, HIV infections, Epidemiology, Population screening

## Abstract

Hepatitis C virus (HCV) contributes to liver-related morbidity and mortality throughout Africa despite effective antivirals. HCV is endemic in the Democratic Republic of the Congo (DRC) but data on HCV/HIV co-infection in pregnancy is limited. We estimated the prevalence of and risk factors for HCV/HIV co-infection among pregnant women in the Kinshasa province of the DRC. This cross-sectional study was conducted as a sub-study of an ongoing randomized trial to assess continuous quality improvement interventions (CQI) for prevention of mother-to-child transmission (PMTCT) of HIV (CQI-PMTCT study, NCT03048669). HIV-infected women in the CQI-PMTCT cohort were tested for HCV, and risk factors were evaluated using logistic regression. The prevalence of HCV/HIV co-infection among Congolese women was 0.83% (95% CI 0.43-1.23). Women who tested positive for HCV were younger, more likely to live in urban areas, and more likely to test positive during pregnancy versus postpartum. HCV-positive women had significantly higher odds of infection with hepatitis B virus (HBV) (aOR 13.87 [3.29,58.6]). An inverse relationship was noted between HCV infection and the overall capacity of the health facility as measured by the service readiness index (SRI) (aOR:0.92 [0.86,0.98] per unit increase). Women who presented to rural, for-profit and PEPFAR-funded health facilities were more likely to test positive for HCV. In summary, this study identified that the prevalence of HCV/HIV co-infection was < 1% among Congolese women. We also identified HBV infection as a major risk factor for HCV/HIV co-infection. Individuals with triple infection should be linked to care and the facility-related differences in HCV prevalence should be addressed in future studies.

## Introduction

Hepatitis C virus (HCV) accounts for a large proportion of morbidity and mortality from liver disease globally, particularly in sub-Saharan Africa (SSA). Recent data suggests that prevalence of HCV in SSA ranges from 0.72% in Southern Africa to 7.82% in Central Africa^1^. A recent national survey in the Democratic Republic of the Congo (DRC) found an HCV prevalence of 0.9%, although prevalence estimates varied by province^2^. Effective antiviral treatment for HCV exists but is not accessible for most inhabitants of SSA, thus perpetuating the epidemic.

Health outcomes in individuals with HCV are far poorer when in the setting of co-infection with HIV. HCV/HIV co-infection has been associated with accelerated liver fibrosis^3^. Liver-related complications have been demonstrated to be a frequent cause of mortality in co-infected individuals as well^4^. Infection with both HCV and HIV has been seen throughout SSA. Several sub-Saharan countries, including Togo, Nigeria, Cameroon, Gabon, Mozambique and the DRC, are considered to be high-prevalence areas (> 3.5% according to published definitions) for HCV/HIV co-infection^5,6^.

Although HCV/HIV co-infection has a clear impact on individuals living in SSA, data on the prevalence of and risk factors for HCV/HIV co-infection in pregnancy are limited. Mother-to-child transmission (MTCT) of HCV is higher among HCV/HIV co-infected mothers compared to HCV mono-infected mothers (10–20% vs. 5–7%, respectively)^7^. It is important to identify HCV/HIV co-infected pregnant women and to determine risk factors for co-infection in order to mitigate this risk and to prevent future infections. We previously conducted a cross-sectional study investigating hepatitis B virus (HBV) and HIV co-infection in pregnant and postpartum women, demonstrating a high prevalence of co-infection and raising concern for increased risk of vertical transmission of both HIV and HBV^8^. Considering the limited data on HCV/HIV co-infection, we expanded upon this previous study to determine the prevalence of and risk factors for HCV-HIV co-infection in Congolese women within the Kinshasa province. We also investigated facility-level factors that were associated with HCV status, such as location and type of facility the women attended, as well as the service readiness index, a measure of the quality of care a facility provides.

## Methods

### Study design and setting

This cross sectional study was a sub-study of an ongoing randomized trial to investigate the effect of data-driven continuous quality interventions (CQI) on long-term anti-retroviral therapies (ART) outcomes among pregnant and breastfeeding women receiving care in Kinshasa province through the Continuous Quality Improvement-Prevention of Mother to Child Transmission (CQI-PMTCT) study, NCT03048669^9^. This study gathered data from 105 sites, all of which were located within the Kinshasa province (Supplementary Fig. S1).

The parent CQI-PMTCT study is prospective in nature, enrolling women at any point from early pregnancy to 12 months postpartum. At enrollment, the HCV status of the eligible participants was assessed. The study was approved by the Ohio State University Institutional Review Board and the Kinshasa School of Public Health Ethical Committee. All methods were carried out in accordance with relevant guidelines and regulations. Pregnant women under the age of 18 were considered emancipated minors in the DRC and were consented directly per the 1987 Law of Emancipation (See Supplementary Information). Written informed consent was obtained from all subjects. This method of consent was approved by the Ethics Committee that reviewed the application. The age of emancipation in the DRC changed to ≥ 18 years in 2019, but enrollment and activities for this study had been completed by that time.

### Inclusion and exclusion criteria

All HIV-infected pregnant or breastfeeding women receiving care at any of the selected MCH facilities between November 2016 and July 2019 were eligible for the parent study, as described previously. Women were excluded if they refused to participate or if they were beyond 12 months postpartum. For this study, women were also excluded if they refused to be tested for HCV.

### Data collection

Eligible participants were referred to study staff for informed consent and enrollment in the study upon presentation for routine visits any time during pregnancy and 12 months postpartum. A participant questionnaire for baseline demographic and clinical data was administered to individuals who consented to be involved in the study. The participants completed survey questions via paper questionnaires administered by the study personnel. These questionnaires were comprised of mostly structured questions, with a few open-ended components. An adapted version of the World` Health Organization (WHO)’s Service Availability and Readiness Assessment (SARA) questionnaire was used to assess the capacity of health facilities to provide general health services^10^.

### Capacity of health facilities to provide general maternal and child health services

Between August and November 2016, we surveyed the 105 sites of enrollment using an adapted version of SARA. Our adapted instrument contained 185 items organized into 12 domains: (1) General information and service availability of the health facility, (2) General service readiness, (3) Staffing, management and quality assurance, (4) Diagnostic services, (5) Antenatal care, (6) PMTCT of HIV infection, (7) Delivery and newborn care services, (8) Well baby clinic, (9) Child curative care services, (10) Family planning services (11), and (12) HIV treatment, care and support.

The service readiness index (SRI) was modeled after the WHO’s service readiness availability index [25], which contains 50 items that are grouped into five categories: (1) basic amenities, (2) basic equipment, (3) infection prevention, (4) diagnostic capacity, and (5) essential medicines (Supplementary Table S1). We identified and matched 35 questions that covered the first four domains—i.e. leaving out the domain of essential medicines. Replicating the WHO’s method, we first computed a percentage score for each domain that is based on the availability of items within a domain. Then, we averaged percentage scores across the four domains to obtain the composite SRI. The Combrach’s alpha (α = 0.77, 95% CI 0.71, 0.83) indicated acceptable internal consistency.

### Outcome variables

The primary outcome of interest for this study was the HCV antibody status of participants (positive or negative). This was measured using the SD Bioline HCV rapid antibody test, verified for use within the region^11,12^.

### Covariates

Other variables considered in this analysis, as in a previous analysis of HBV/HIV co-infection, included participants’ socio-demographic information, clinical features and health facility characteristics. Participant socio-demographic characteristics included the following: maternal age (≤ 24, 25–34, ≥ 35), marital status (married/cohabitating vs. divorced/separated/widowed/never married), educational level (primary, secondary, or tertiary), alcohol consumption (no vs. yes), and socioeconomic status (SES) - measured by a wealth index score. Calculation of the wealth index score is described by Mpody et al.^13^.

Clinical characteristics included: HBV infection (yes vs. no – using the Alere HBsAg rapid test)^14^, duration of ART in months (≤ 6, 6–24, or > 24), disclosure of HIV status to anyone (yes vs. no), primigravida (yes vs. no), report of any intimate partner violence (IPV; yes vs. no) and HIV virological suppression [Viral load (VL) ≤ 1000 copies/mL (suppressed) vs. VL > 1000 copies/mL (unsuppressed)].

Facility factors included location (urban vs. peri-urban/rural), type of facility (hospital vs. health center, as per self-report by the facility manager), and whether they receive direct PEPFAR funding support for HIV care (yes vs. no). Of note, maternity clinics in Kinshasa receive funding for the provision of HIV care either from PEPFAR or from the Global Fund. Non-HIV care is provided via fee-for-service care across the various types of facilities.

### Statistical analysis

Statistical analyses were similar to those of a previous study of HBV/HIV co-infection in the same population^8^. We calculated the proportion of participants with a positive HCV antibody test as a measure of HCV prevalence with associated 95% binomial confidence intervals (CIs). Bivariate and multivariable logistic regression modeling was used to estimate prevalence odd ratios (OR) and Wald’s 95% CI comparing the prevalence of HCV antibody across levels of covariates. Covariates were included in the multivariable model if the p-value was < 0.20 in bivariate analysis. Generalized estimating equation was used to account for potential clustering at the level of health facilities. Only participants with complete data on all covariates were included in the final analysis. All analyses were conducted using SAS, version 9.3 (SAS Institute Inc., Cary, North Carolina), and STATA/IC version 14.0 (StataCorp LP, College Station, Texas). All tests, unless otherwise indicated, were conducted using a two-sided 0.05 significance level, without correction for multiple comparisons.

## Results

**Figure 1:**
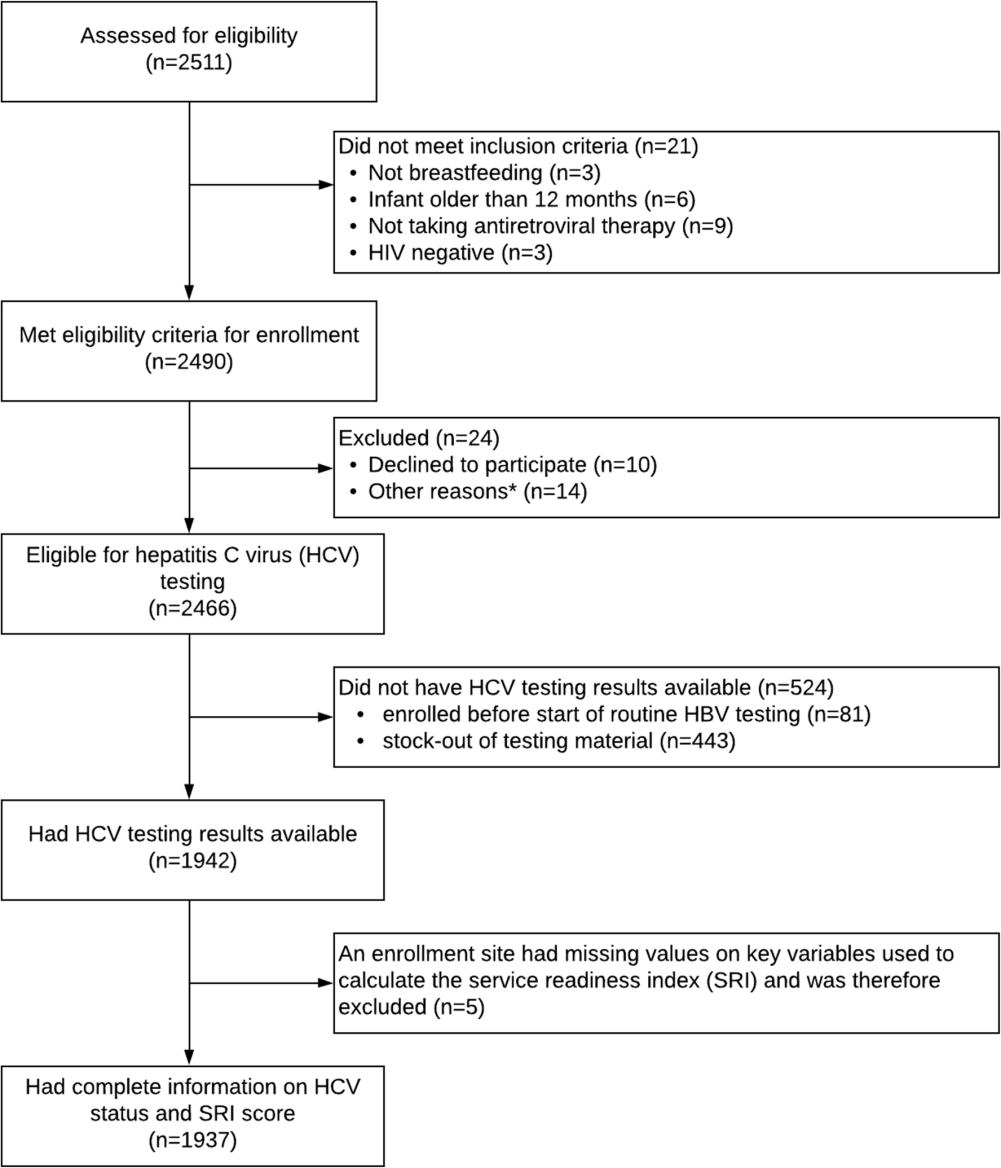
Flow chart of eligibility and enrollment.

Of 1,942 HIV-positive Congolese women tested for HCV, 16 (0.83%; 95% CI 0.43-1.23) were HCV/HIV co-infected (Fig. 1). The majority (93.0%) of women who were tested attended facilities in urban areas and most (57.6%) were enrolled in a hospital setting (Table 1). Most women were under 35 years of age, with over half (52.7%) between 25–34 years of age, and 31.1% being 24 years or younger. The youngest participant was 15 years of age and the oldest 50 years of age. About two-thirds (67.8%) of women included in the parent study were either divorced, separated, widowed, or never married. Very few (1.9%) of the women in the study were smokers, and the majority (72.4%) of women denied any alcohol consumption. The majority (84.4%) of women had at least a secondary education. HIV RNA viral load in most (64.1%) women was less than 1000 copies/mL. About half of the women involved in the study had disclosed their HIV status. The HBV prevalence in this cohort was 4.2%.

**Table 1:** Sociodemographic, clinical and facility characteristics of the study population.

	No(%) n = 1942
**Study participant characteristics**	
**Age**	
35+	305(16.1)
25–34	997(52.7)
≤ 24	589(31.1)
Missing	51
**Timing of testing**	
Pregnancy	1154(59.4)
Delivery	417(21.5)
Post-partum	371(19.1)
Missing	0
**Marital status**	
Married/cohabitating	608(32.2)
Divorced/separated/widow/never married	1283(67.8)
Missing	51
**Alcohol consumption**	
No	1369(72.4)
1–3/month	416(22)
> 2/week	106(5.6)
Missing	51
**Smoking status**	
Previous/current	36(1.9)
Never	1846(98.1)
Missing	60
**Educational level**	
Tertiary	245(13)
Secondary	1348(71.4)
Primary	296(15.7)
Missing	53
**SES in tertile**^¶^	
3 (Highest)	607(33.4)
2	608(33.5)
1(Lowest)	600(33.1)
Missing	127
**Primigravida**	
Yes	195(10.3)
No	1700(89.7)
Missing	47
**Any intimate partner violence**^#^	
No	1196(63.2)
Yes	696(36.8)
Missing	50
**HIV RNA viral load**	
≥ 1000 copies/mL	678(35.9)
< 1000 copies/mL	1208(64.1)
Missing	56
**ART regimen**	
TDF + 3TC + FEV	1601(82.4)
AZT + 3TC + NVP	201(10.4)
Other	140(7.2)
**Duration of ART**	
< 12 months	846(45)
13–24 months	215(11.4)
≥ 24 months	819(43.6)
Missing	62
Disclosure of HIV status**	
No	910(47)
Yes	1028(53)
Missing	4
**HBV status**	
Negative	1858(95.8)
Positive	81(4.2)
Missing	3

	No (%) n = 1942
**Facility characteristics**^§^	
**Location of facility attended**	
Peri-urban/rural	135(7)
Urban	1802(93)
Missing	5
**Type of facility of care**	
Health center	821(42.4)
Hospital	1116(57.6)
Missing	5
**PEPFAR funding**	
No	731(37.7)
Yes	1206(62.3)
**Type of facility of care**	
Public	623(32.2)
Private for profit	249(12.9)
Private non-profit	1065(55)

The analytical sample was derived from the enrollment data of an ongoing cluster randomized controlled trial, aimed at evaluating the effect of data-driven continuous quality improvement on long-term ART outcomes in Kinshasa, Democratic Republic of Congo. We retained participants that had available data on HCV rapid testing.

SES, socio-economic status; RNA, ribonucleic acid, ART, antiretroviral therapy, HBV, hepatitis B virus.

**Self-report of disclosure of HIV status to anyone.

^§^Facility at which participant was enrolled/tested.

^¶^Calculated using principal component analysis and categorized in three groups: the lower first two quintiles, the middle quintiles, and the last two quintiles.

^#^Self-report of emotional or physical or sexual partner violence.

Results of the bivariable and multivariable analyses are shown in Tables 2 and 3, respectively. Regarding individual characteristics, none of the HCV-positive women reported any smoking history, and the majority of women denied any alcohol consumption. In bivariable analyses, there was an association between testing positive for HBV and HCV (uOR 7.79 [2.37,25.59]). In multivariable analyses, this association with triple infection held true: the prevalence of HCV was substantially higher among women infected with hepatitis B compared to those who tested negative for HBV (4.9% vs 0.6%, aOR 13.87 [3.29,58.6]). Although not statistically significant in multivariable analyses, the prevalence of HCV was higher among women who had been on anti-retroviral therapy for less than 12 months compared to those with greater than 24 months of ART (1.3% vs. 0.5%, aOR 0.49 [0.14,1.66]). Regarding facility characteristics, women attending private for-profit (uOR 6.38 [1.15,35.42]; aOR 19.18 [2.00,183.53]) were more likely to test positive for HCV than those attending public facilities. Additionally, women attending facilities in urban areas were less likely to test positive for HCV as opposed to those attending facilities in peri-urban/rural areas (aOR 0.35 [0.07,1.84]). Women who attended facilities with PEPFAR funding (aOR 6.72 [1.35,33.42]) or facilities with lower capacity to provide general maternal and child health services were also more likely to test positive for HCV. Each percent point increase in the SRI was associated with an 8% lower odds in HCV prevalence (aOR:0.92 [0.86,0.98]), relatively.

**Table 2:** Bivariable associations between sociodemographic, clinical and facility characteristics of the study population.

HCV+	n/N(%)	uOR(95% CI)^†^	P value
**Service readiness index**		0.95(0.90,0.99)	0.019
**Location of facility** ^§^ ** attended**			
Peri-urban/rural	2/135(1.5)		
Urban	14/1802(0.8)	0.56(0.10,2.96)	0.492
**Type of facility of care**			
Health center	8/821(1.0)		
Hospital	8/1116(0.7)	0.72(0.25,2.07)	0.544
**PEPFAR funding**			
No	3/731(0.4)		
Yes	13/1206(1.1)	2.62(0.69,9.88)	0.156
**Type of facility of care**			
Public	2/623(0.3)		
Private for profit	5/249(2.0)	6.38(1.15,35.42)	0.034
Private non-profit	9/1065(0.8)	2.76(0.55,13.85)	0.217
**Age**			
≤ 24	3/305(1.0)		
25–34	6/997(0.6)	0.62(0.15,2.52)	0.502
35+	7/589(1.2)	1.22(0.31,4.82)	0.778
**Timing of testing**			
Pregnancy	12/1154(1)		
Delivery	4/417(1.0)	0.91(0.29,2.88)	0.878
Post-partum	0/371(0)	–	–
**Marital status**			
Divorced/widowed/never married	6/608(1.0)		
Married/cohabitating	10/1283(0.8)	0.79(0.28,2.20)	0.646
**Alcohol consumption**			
No	10/1369(0.7)		
1–3/month	5/416(1.2)	1.60(0.53,4.81)	0.399
> 2/week	1/106(0.9)	1.20(0.14,10.33)	0.869
**Smoking**			
Previous/current	0/36(0.0)		
Never	16/1846(0.9)	–	–
**Educational level**			
Primary	3/245(1.2)		
Secondary	13/1348(1)	0.77(0.22,2.72)	0.685
Tertiary	0/296(0.0)	–	–
**SES in tertile** ^¶^			
1(Lowest)	4/607(0.7)		
2	7/608(1.2)	1.84(0.53,6.43)	0.340
3 (Highest)	4/600(0.7)	1.04(0.25,4.28)	0.960
**Primigravida**			
Yes	1/195(0.5)		
No	15/1700(0.9)	1.82(0.22,14.65)	0.576
**Any intimate partner violence** ^#^			
No	10/1196(0.8)		
Yes	6/696(0.9)	0.97(0.34,2.74)	0.957
**HIV RNA viral load**			
≥ 1000 copies/mL	8/678(1.2)		
< 1000 copies/mL	7/1208(0.6)	0.49(0.18,1.36)	0.170
**Duration of ART**			
< 12 months	11/846(1.3)		
13–24 months	1/215(0.5)	0.34(0.04,2.74)	0.311
≥ 24 months	4/819(0.5)	0.37(0.12,1.17)	0.089
Disclosure of HIV status**			
No	9/910(1.0)		
Yes	7/1028(0.7)	0.69(0.25,1.87)	0.467
**HBV**			
No	12/1858(0.6)		
Yes	4/81(4.9)	7.79(2.37,25.59)	0.001
**ART regimen**			
TDF + 3TC + FEV	15/1601(0.9)		
AZT + 3TC + NVP	1/201(0.5)	0.50(0.06,4.13)	0.523
Other	0/140(0.0)	–	–

^*^The analytical sample was derived from the enrollment data of an ongoing cluster randomized controlled trial, aimed at evaluating the effect of data-driven continuous quality improvement on long-term ART outcomes in Kinshasa, Democratic Republic of Congo. We retained participants that had available data on HCV rapid testing.

SES, socio-economic status; RNA, ribonucleic acid; ART, antiretroviral therapy; HBV, hepatitis B virus; uOR, unadjusted odds ratio; aOR, adjusted odds ratio; 95%CI, 95% confidence interval.

^§^Facility at which participant was enrolled/tested.

^¶^Calculated using principal component analysis and categorized in three groups: the lower first two quintiles, the middle quintiles, and the last two quintiles. #Self-report of emotional or physical or sexual partner violence.

**Self-report of disclosure of HIV status to anyone.

^†^OR and 95%CI were obtained using logistic models and generalized estimating equation to adjust for potential clustering at the level of the clinic.

**Table 3:** Multivariable associations between sociodemographic, clinical and facility characteristics of the study population.

	aOR(95% CI)^†^ Individual characteristics	P value	aOR(95% CI) Facility characteristics^§^	P value	aOR(95% CI) Combined characteristics	P value
**Service readiness index**	0.94(0.89,0.99)	0.017	0.94(0.89,0.99)	0.021	0.92(0.86,0.98)	0.010
**Location of facility attended**						
Peri-urban/rural						
Urban			0.47(0.11,2.02)	0.309	0.35(0.07,1.84)	0.216
**Type of facility of care**						
Health center						
Hospital			1.17(0.39,3.50)	0.782	1.05(0.28,3.93)	0.944
**PEPFAR funding**						
No						
Yes			2.93(0.89,9.69)	0.078	6.72(1.35,33.42)	0.020
**Type of facility of care**						
Public						
Private for profit			6.40(1.35,30.22)	0.019	19.18(2.00,183.53)	0.010
Private non-profit			2.65(0.64,11.03)	0.180	7.15(0.83,61.68)	0.074
**Age**						
≤ 24						
25–34	0.49(0.10,2.33)	0.369			0.50(0.10,2.55)	0.404
35+	1.41(0.31,6.48)	0.656			1.51(0.30,7.54)	0.612
**Timing of testing**						
Pregnancy						
Delivery	0.74(0.20,2.75)	0.652			0.62(0.16,2.39)	0.49
Post-partum	–	–			–	–
**SES in tertile** ^¶^						
1(Lowest)						
2	1.71(0.47,6.18)	0.415			1.74(0.45,6.68)	0.419
3 (Highest)	1.53(0.35,6.78)	0.573			1.90(0.41,8.91)	0.416
**Primigravida**						
Yes						
No	2.16(0.23,20.21)	0.498			1.95(0.20,19.25)	0.566
**Duration of ART**						
< 12 months						
13–24 months	–	–			–	–
≥ 24 months	0.44(0.13,1.46)	0.180			0.49(0.14,1.66)	0.249
**HBV**						
No						
Yes	12.07(3.31,44.05)	< 0.001			13.87(3.29,58.6)	0.001

The analytical sample was derived from the enrollment data of an ongoing cluster randomized controlled trial, aimed at evaluating the effect of data-driven continuous quality improvement on long-term ART outcomes in Kinshasa, Democratic Republic of Congo. We retained participants that had available data on HCV rapid testing.

SES, socio-economic status; RNA, ribonucleic acid; ART, antiretroviral therapy; HBV, hepatitis B virus; uOR, unadjusted odds ratio; aOR, adjusted odds ratio; 95%CI, 95% confidence interval.

^§^Facility at which participant was enrolled/tested.

^¶^Calculated using principal component analysis and categorized in three groups: the lower first two quintiles, the middle quintiles, and the last two quintiles.

^†^OR and 95%CI were obtained using logistic models and generalized estimating equation to adjust for potential clustering at the level of clinic.

## Discussion

The prevalence of HCV/HIV co-infection among pregnant and postpartum women living in Kinshasa Province in the DRC was 0.83%. This is consistent with prior estimates of HCV infection in the DRC, irrespective of HIV status^2^. This study also found that the odds of HCV infection was 13.87 times higher in HBV-infected women than HBV-uninfected women, raising concerns for a vulnerable population of HCV/HBV/HIV triple-infected women.

Factors contributing to triple-infection are not entirely clear and studies investigating outcomes in those with triple infection of HCV/HBV/HIV are limited. A recent study in Nigeria found that in those with HCV/HBV/HIV triple-infection, CD4+ counts are significantly lower as compared to those with HIV infection alone. Individuals with triple-infection also achieve less benefit with anti-retroviral therapy than those with mono-HIV infection^15^.

Effective treatments for HCV are available but are not easily accessible for most inhabitants of the DRC. While MTCT of HCV is relatively low (5–7%) in HCV mono-infected mothers, rates of MTCT of HCV are higher (10–20%) among HCV/HIV co-infected women^7^. However, HCV treatment is not recommended during pregnancy and effective interventions to prevent MTCT in HCV-infected pregnant women have yet to be identified. HCV-infected infants are subsequently at high risk for liver disease progression^16^. It is therefore of utmost importance that future efforts focus on the treatment of HCV-infected women of childbearing age *prior to pregnancy* in African settings. In addition, measures for PMTCT of HIV and HBV should be prioritized in cases of HCV/HBV/HIV triple infection to reduce the chances of these infections in women and their infants.

This study highlights facility-level, socio-demographic and clinical factors associated HCV/HIV co-infection in pregnant and breastfeeding women. HCV/HIV co-infected women were more likely to present for care during pregnancy than in the postpartum period, which argues that pregnant women should be targeted for screening during a time when they are more likely to seek care. HCV/HIV co-infected women were also younger and more likely to live in urban areas. We found that women who attended private facilities in general, and for-profit facilities in particular, were more likely to be HCV-positive. The primary route of HCV transmission in African settings is via nosocomial spread, and thus facility characteristics (sterilization of equipment, medical procedures performed, etc.) are important to examine in depth in future studies^17^. As expected, women attending facilities with a higher capacity to provide general maternal and child health services (high SRI score) were less likely to be infected with HCV. We also found that HCV-positive women were more likely to attend facilities with direct PEPFAR funding. PEPFAR supported facilities are typically large urban hospitals and generally provide better HIV care services^13^, thus it is possible that this association is due to residual confounding.

The strength of this study lies in its contribution to a field with a current paucity in data. There is very little research available investigating HIV/HCV co-infection in pregnant Congolese women, making this study one of the first to evaluate this specific population. This study also considers both typical HCV risk factors as well as factors specific to the facilities in which women seek care.

A primary limitation of this study was its generalizability, as data was obtained from pregnant women only within Kinshasa Province and therefore may not generalizable outside of this region. Additionally, as with any cross-sectional study, we were not able to imply causation. This study was also limited by a lack of confirmatory HCV RNA testing, with only HCV antibody status testing evaluated. Thus, we were not able to decipher timing of infection (active vs. past). An additional limitation of this study was the testing method used to evaluate HCV status in the patient population. Although the Abbott Determine HCV test has been verified in other settings (in Nigeria, Georgia, Cambodia, and Belgium as referenced), no data is available regarding its analytical performance in the DRC.

## Conclusion

The prevalence of HCV/HIV co-infection among pregnant Congolese women in this study was consistent with previous estimates of HCV prevalence in the region. HCV/HIV co-infected women tended to be younger, live in urban areas, and seek care in private and PEPFAR-funded clinics. Facility-related risk factors should be further researched, as nosocomial transmission is a major risk factor for HCV infection in African settings. HCV/HIV co-infected women had a much higher odds of being triple-infected with HBV as well, a phenomenon that warrants further investigation. Future studies should target women during pregnancy for HCV screening in order to implement curative treatment post-pregnancy and thus prevent transmission in subsequent pregnancies.

## Supplementary Information

Supplementary Information.
