# Supplementary material for: Hepatitis C prevalence and quality of health services among HIV-positive mothers in the Democratic Republic of the Congo
Source: Sci Rep. 2022 Jan 26;12:1384. doi: 10.1038/s41598-022-05014-3 (PMC8791992; doi:10.1038/s41598-022-05014-3)
Supplement: Supplementary file 1 — Supplementary Information. [file 41598_2022_5014_MOESM1_ESM.docx]

**Supplementary Materials**

**Hepatitis C Prevalence and Quality of Health Services among HIV-positive Mothers in the Democratic Republic of the Congo**

Short Title: **HCV/HIV Co-infection in Congolese Mothers**

**Supplementary Figure:**


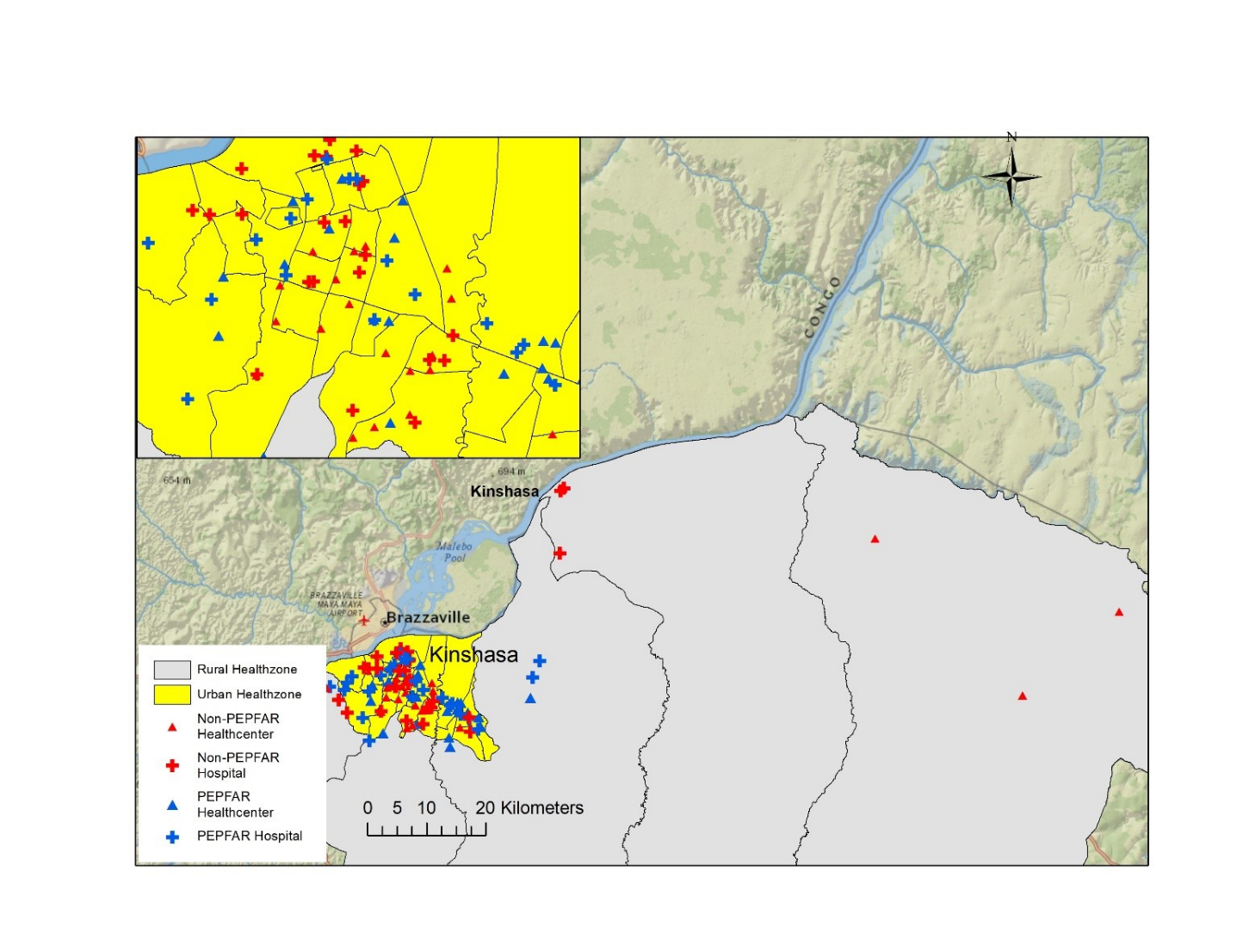


**Supplementary Figure 1.** Map of Kinshasa showing the sites of enrollment of patients and their characteristics ^1,2,3^

**Supplementary Table:**

**Supplementary Table 1**: Components of SRI; Kinshasa facility survey, 2016*

| Characteristic | Overall | | Hospitals | | Health Centers | |
| --- | --- | --- | --- | --- | --- | --- |
|  | (n=105) | | (n=54) | | (n=51) | |
|  | No | % † | No | % † | No | % † |
| **Basic Amenities** |  |  |  |  |  |  |
| Telephone | 43 | (41) | 27 | (50) | 16 | (31) |
| Computer | 56 | (53) | 40 | (74) | 16 | (31) |
| Access to Email or Internet | 23 | (22) | 18 | (33) | 5 | (10) |
| Electricity | 91 | (87) | 51 | (94) | 40 | (78) |
| Running Water | 94 | (90) | 49 | (91) | 45 | (88) |
|  |  |  |  |  |  |  |
| **Basic amenities full comprehensive‡** | **0** | **(0)** | **0** | **(0)** | **0** | **(0)** |
|  |  |  |  |  |  |  |
| **Basic equipment** |  |  |  |  |  |  |
| Thermometer | 94 | (94) | 49 | (92) | 45 | (96) |
| Digital BP Apparatus | 87 | (83) | 47 | (87) | 40 | (78) |
| Manual BP Apparatus | 101 | (96) | 51 | (94) | 50 | (98) |
| Stethoscope | 105 | (100) | 54 | (100) | 51 | (100) |
| Fetal Stethoscope | 105 | (100) | 54 | (100) | 51 | (100) |
| Examination Light | 100 | (95) | 51 | (94) | 49 | (96) |
| Adult Weighing Scale | 105 | (100) | 54 | (100) | 51 | (100) |
| Child Weighing Scale | 69 | (66) | 38 | (72) | 31 | (61) |
|  |  |  |  |  |  |  |
| **Basic equipment full comprehensive§** | **34** | **(32)** | **23** | **(43)** | **11** | **(22)** |
|  |  |  |  |  |  |  |
| **Infection Prevention** |  |  |  |  |  |  |
| Hand-Washing Soap | 91 | (87) | 50 | (93) | 41 | (80) |
| Alcohol-Based Hand Rub | 57 | (54) | 32 | (59) | 25 | (49) |
| Waste Receptacle with Lid and Liner | 68 | (65) | 37 | (69) | 31 | (61) |
| Other Waste Receptacle | 92 | (88) | 50 | (93) | 42 | (82) |
| Sharps Container | 93 | (89) | 49 | (91) | 44 | (86) |
| Disposable Latex Gloves | 98 | (93) | 51 | (94) | 47 | (92) |
| Disinfectant | 77 | (73) | 45 | (83) | 32 | (63) |
|  |  |  |  |  |  |  |
| **Infection prevention full comprehensive¶** | **53** | **(51)** | **23** | **(45)** | **11** | **(22)** |
|  |  |  |  |  |  |  |
| **Diagnostic capacity** |  |  |  |  |  |  |
| Tuberculosis Testing On-Site | 61 | (58) | 36 | (67) | 25 | (49) |
| Dip Sticks used for Urine Glucose | 68 | (65) | 43 | (80) | 25 | (49) |
| Syphilis Testing On-Site | 56 | (53) | 35 | (65) | 21 | (41) |
| Syphilis Rapid Diagnostic Test | 45 | (43) | 31 | (57) | 14 | (27) |
| Any Rapid Test for Hemoglobin | 94 | (90) | 50 | (93) | 44 | (86) |
| HemoCue Present | 33 | (31) | 20 | (37) | 13 | (25) |
| Facility Completes Stool Microscopy | 104 | (99) | 54 | (100) | 50 | (98) |
| Urine Pregnancy Test | 92 | (88) | 50 | (93) | 42 | (82) |
| HIV rapid diagnostic testing | 101 | (96) | 53 | (98) | 48 | (94) |
| Urine Protein Test | 84 | (80) | 50 | (93) | 34 | (67) |
| Urine Glucose Test | 85 | (81) | 51 | (94) | 34 | (67) |
| Malaria Rapid Diagnostic Testing | 52 | (50) | 25 | (46) | 27 | (53) |
| Diagnostic X-rays Performed On-Site | 43 | (41) | 35 | (65) | 8 | (16) |
| Ultrasound Performed On-Site | 71 | (68) | 46 | (85) | 25 | (49) |
|  |  |  |  |  |  |  |
| **Diagnostic capacity full comprehensive#** | **7** | **(7)** | **7** | **(13)** | **0** | **(0)** |
|  |  |  |  |  |  |  |

*This is survey of 105 health facilities across the 35 health districts of Kinshasa conducted between August 2016 and May 2017†Column percentage. Abbreviations: PMTCT, Prevention of mother to child transmission of HIV; ANC, Antenatal care; ART, Antiretroviral therapy. ‡Assesses whether a facility attained full comprehensiveness in terms of availability of items the domain of “Basic amenities”. §Assesses whether a facility attained full comprehensiveness in terms of availability of items in the domain of “Basic equipment”. ¶Assesses whether a facility attained full comprehensiveness in terms of availability of items in the domain of “Infection prevention”. #Assesses whether a facility attained full comprehensiveness in terms of availability of items in the domain of “Diagnostic capacity”. Abbreviations; BP, blood pressure.

**References**

1. Anokwa, Y., Hartung, C., Brunette, W., Borriello, G. & Lerer, A. Open source data collection in the developing world. 42(10) (2009).

2. Hartung, C. *et al.* Open data kit: Tools to build information services for developing regions. *ACM Int. Conf. Proceeding Ser.* 1–12 (2010) doi:10.1145/2369220.2369236.

3. Esri Inc. (1999). ArcGIS (Version 10.2). Esri Inc. https://www.esri.com/en-us/arcgis/products/arcgis-online/overview.
